# Integrating Ultrasound-Derived Carotid Artery Stiffness in the Assessment of Cardiovascular Risk

**DOI:** 10.33425/2639-8486.1172

**Published:** 2023-06-22

**Authors:** J Ryan Gedney, Jean Marie Ruddy

**Affiliations:** Medical University of South Carolina, Division of Vascular Surgery, Charleston, South Carolina.

**Keywords:** Arterial stiffness, Speckle tracking, Vector velocity imaging, Carotid ultrasound, Cardiovascular risk

## Abstract

Historically, pulse wave velocity (PWV) has been used to measure vascular stiffness, but is limited in its utility when certain vascular disease states are present, such as aneurysm or iliac stenosis. PWV can therefore only provide reliable assessment of global vascular stiffness in limited vascular pathology. Speckle tracking is a method of post-hoc ultrasound image analysis that can measure vascular stiffness in a more comprehensive manner. Evidence from in vitro as well as in vivo studies has validated these techniques in the assessment of strain, distensibility, modulus, and stiffness index (β) in the carotid arterial system. Unfortunately, despite the well-established correlation between vascular stiffness and cardiovascular morbidity and mortality, standard vascular laboratory ultrasound protocols do not include stiffness assessment. Herein, we present evidence in favor of integrating speckle tracking into carotid artery duplex protocols to measure vascular stiffness that can be utilized in medical management to modulate cardiovascular risk.

## Introduction

Vascular stiffening results from alterations in the cellular and extracellular elements of the vessel wall due to age, hemodynamic forces, inflammation, and pathologic cell signaling, and is a biomarker of increased risk for cardiovascular morbidity and mortality [1–8] Evidence from the Rotterdam study demonstrated increased aortic stiffness was an independent predictor of heart disease and stroke in patients that otherwise were healthy [7]. Arterial stiffness has also demonstrated significant direct relationships with myocardial infarction, heart failure, and all-cause mortality [1,4–6].

Vascular stiffening may be clinically recognized as an increase in systolic blood pressure and widening of pulse pressure [8], but pulse wave velocity (PWV) has been the most common means of measuring vascular stiffness and can demonstrate increased values with age related development of arteriosclerosis [2,9]. Cardiovascular risk and all-cause mortality, in particular, increase as much as 15% each with a 1 m/s elevation in PWV [5,10]. Quantification of PWV can be compromised by several common cardiovascular disease states, including AAA and peripheral arterial occlusive disease [11,12], so pursuance of a more reliable technique, with fewer confounding comorbidities, can amplify access to vascular stiffness measurements and potentially direct medical therapy titration in clinical scenarios known to promote stiffness, such as hypertension.

Speckle tracking is a tool involved in ultrasound image analysis and can directly measure vascular stiffness [2,13,14]. This tool overcomes the limitations of PWV by measuring local vascular stiffness, even when pathology is present [15]. Speckle tracking has been investigated as a tool in measuring vascular stiffness and its relationship to different disease states, including aging [16–18], hypertension [19,20], diabetes [20], pre-eclampsia [21], Takayasu’s arteritis [22], and atherosclerosis [23,24]. Speckle tracking also has substantial evidence for its use in the carotid artery [25–28], with evidence directly involving the use of the technology in the carotid artery in the presence of vascular disease [24,27,29]. Increased vascular stiffness in this setting is identified as abnormal measurements of circumferential strain, elastic modulus, distensibility, compliance, and stiffness index [24]. This offers the potential to integrate speckle tracking and these stiffness metrics directly into already existed carotid ultrasound protocols as an evidence based tool.

Stiffness of the carotid artery has a direct relationship to cardiovascular morbidity and mortality [30]. An elastic modulus of greater than 1 MPa has significantly higher cardiovascular mortality out to 25 months [31,32]. Other important investigations (Table 1) that use ultrasound to measure local carotid artery stiffness parameters have shown increased carotid artery stiffness in patients with coronary artery disease [33,34], diastolic heart dysfunction [35], and those with cardiovascular inflammatory markers, CRP and BNP [36], demonstrating the increased cardiovascular risk of patients with elevated carotid artery stiffness. The objective of this review is to promote the use of speckle tracking for vascular stiffness measurement as an integral component of routine carotid artery ultrasound protocols, with the goal of offering a more comprehensive assessment of a patient’s cardiovascular risk.

## Assessing Vascular Stiffness

Ultrasound is already an integral component in the diagnosis, management, and surveillance of carotid artery disease. Protocols capture images in B-mode to assess arterial morphology, Color Doppler to visualize laminar versus turbulent flow, and Doppler waveforms for a spectral analysis of flow velocity across the cardiac cycle [37]. The ability to visualize dynamic changes to vessel wall dimensions is utilized to calculate stiffness metrics.

M-mode vessel diameters may be measured across the cardiac cycle, collecting corresponding points of systolic and diastolic blood pressure to generate a pressure-diameter relationship that facilitates quantification of stiffness metrics [15,38–43]. Applying this concept to the carotid artery, investigators were able to capture distensibility, compliance, pressure-strain elastic modulus, and Young’s Modulus in a series of healthy patients [38]. Age-related changes in each parameter corresponded to increased vascular stiffness [38]. Similar methods of manual M-mode calculations of vascular stiffness have demonstrated a correlation to post-mortem findings of stiffness [41–43]. In such a study, stiffness index measured via M-mode diameter changes relative to blood pressure strongly correlated with the atherosclerotic grade measured via post-mortem histological analysis [41]. Ultimately, these techniques provide a mathematical backbone to calculate stiffness parameters via ultrasound, but they require manual measurements that limit seamless integration into current ultrasound protocols.

## Speckle Tracking

**Figure 1:**
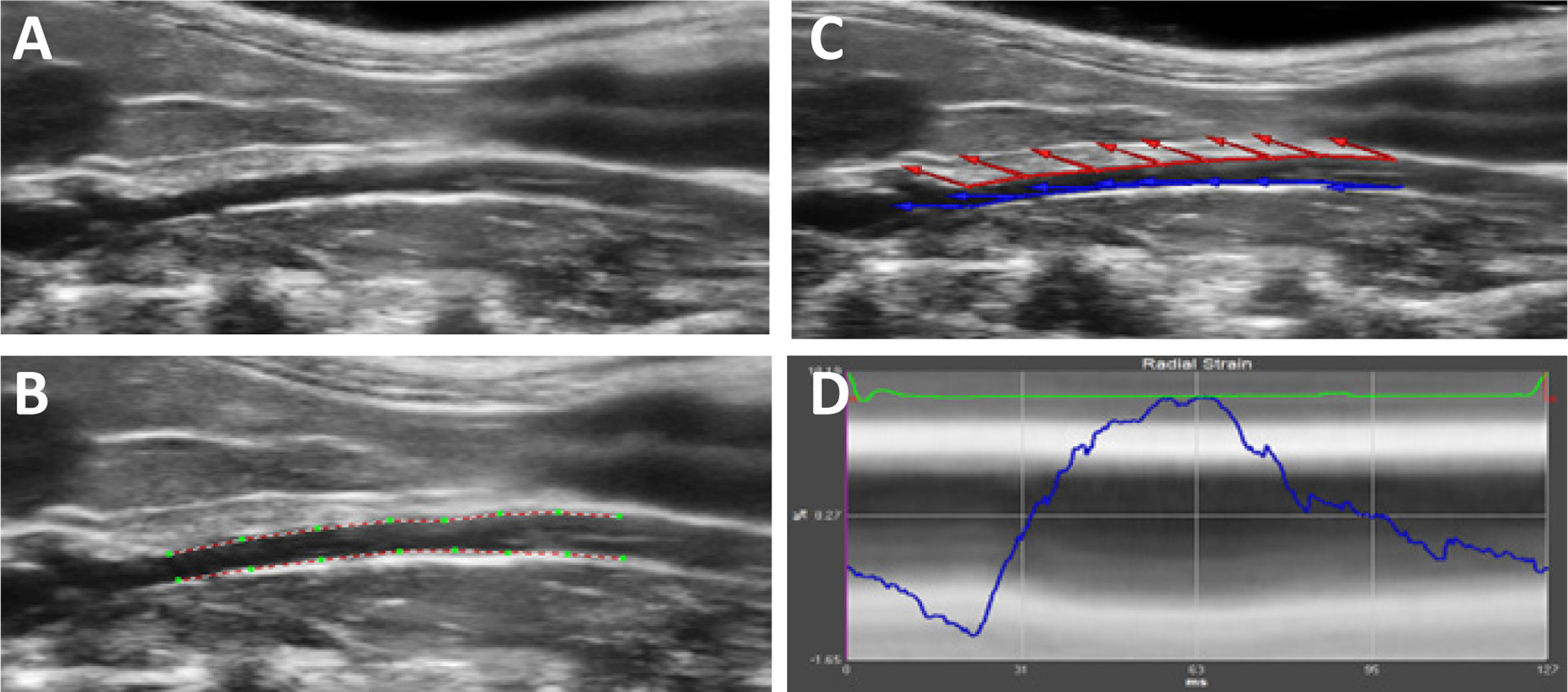
Images representing speckle tracking with vector velocity imaging of the murine abdominal aorta: (A) longitudinal view of the artery in Bmode, (B) speckle set-up to track the walls of the artery, (C) vector velocity images as the blood vessel expands in systole, and (D) the strain curve output across the cardiac cycle.

Speckle tracking is a form of semi-automated ultrasound image analysis that can be used offline after image acquisition to assess vascular wall mechanics, and ultimately calculate any stiffness parameters without the need for manual calculations. Software is used to identify acoustic tissue markers within the ultrasound images and track across the duration of the ultrasound image. Speckles are identified along the vessel wall, and motion is tracked independent of any other variable, thereby enabling analysis of ultrasound images and vascular wall motion in an angle-independent manner (Figure 1) [2,14,24]. Vector velocity imaging is a software-integrated tool used in speckle tracking ultrasound image analysis to evaluate multiple directions of deformation at the same time, and has proven particularly advantageous in the carotid artery and the aorta [13,15]. The technique plots the location of a designated ‘kernel’ within a region of interest across multiple ultrasound images over time. As the location of the kernel is tracked across images relative to the same region of interest, a multidirectional analysis of deformation across the cardiac cycle can be constructed [44]. This technology allows for the evaluation of discrete kernels or segments of kernels relative to one another, increasing the analysis power of the tool [18,25]. Studies validating an optimal frame rate of 60 to 110 frames per second have been performed with measurements done in both the long and short axis of carotid arteries [14]. Using vector mathematics, the displacement of kernels can be calculated to produce strain values [27], and stiffness parameters may be derived in different directions, including radial, circumferential, and longitudinal.

Speckle tracking can also be beneficial because it enables quantification of multiple mechanical parameters with defined stiffness relationships, all from a single image dataset, providing comprehensive analysis of vascular remodeling. Vessel wall strain can be represented as a percent change in diameter between systole and diastole, therefore strain decreases with increased vascular stiffness [2,23,25]. Integrating pressure to derive the stiffness index (β) can be calculated by taking the natural log of the ratio between the systolic and diastolic blood pressure, and then dividing by the circumferential strain [2,23]. Carotid artery circumferential strain and stiffness index demonstrate a clear negative correlation such that increases in arterial stiffness result in decreased circumferential strain [13]. Distensibility represents the strain per unit pressure and is often derived by dividing strain by pulse pressure [2,23,25]. Like strain, distensibility will also decrease with increased vascular stiffness, as a stiffer artery will displace less under constant pressure conditions. Elastic modulus is an inherent mechanical property used in material science that can be applied to vessel wall mechanics, described as stress divided by strain, that can be calculated by the change in pressure over the change in diameter between two points, often across the cardiac cycle [2,23]. Because of this multi-functionality, speckle tracking is well-suited for application to various vascular structures in the assessment of stiffness.

## Applying Speckle Tracking in Carotid Artery Ultrasound

The carotid artery is an accessible, easy to evaluate arterial bed where ultrasound is currently utilized to detect atherosclerotic plaque, but substantial evidence supports the application of ultrasound derived stiffness parameters to the carotid artery [27]. For instance, stiffness metric validation studies comparing carotid artery duplex in human subjects to known standard controls of polyvinyl models perfused by pulsatile pumps have demonstrated that speckle tracking can effectively estimate strain against a standard in the short-axis plane [26]. Furthermore, speckle-tracking analysis of ultrasound images obtained on the polyvinyl phantom models was similar to the reference strain applied to the model measured by sonomicrometry, suggesting the use of carotid speckle tracking in human subjects is feasible [26].

Ultrasound derived stiffness parameters are able to identify subclinical atherosclerosis [24,45]. This is extremely important to appropriate risk stratification in healthy patients, and speckle tracking derived stiffness metrics can see changes in vascular stiffness of the carotid artery even in this population [18]. Circumferential strain normalized to pulse pressure is significantly different between cardiovascular risk groups and is effective at identifying early signs of vascular disease in healthy individuals at low cardiovascular risk [23]. Additionally, modulus better determines differences between healthy and hypertensive patients than PWV [18,28], and had less inter-observer variability than stiffness index, which is manually calculated as opposed to speckle tracked [28]. Other evidence concludes that circumferential strain measured via speckle tracking is better than manual measurements of stiffness because it accounts for the entire circumferential movement of the wall, instead of just diameter changes [17]. Furthermore, ultrasound derived carotid stiffness parameters using speckle tracking are also automated and significantly reproducible [23], simply requiring analysis of transverse or longitudinal B-mode image loops. Overall, patients with risk factors for subclinical atherosclerosis appear to benefit more from stiffness assessment via speckle tracking than other forms of risk stratification.

As previously stated, vascular stiffness in patients with risk factors for arterial disease, including age, hypertension, coronary artery disease, and diabetes, has been studied as a means of evaluating cardiovascular risk [20,23,27–29]. For example, arterial stiffness in the aging carotid artery has been measured via speckle tracking and demonstrated clear decreased strain values in older individuals [17]. Additionally, hypertensive patients have demonstrated lower carotid strain and higher stiffness index than sex and age matched healthy controls at a single time point [20,28], and lower peak carotid artery strain has been detected in diabetic patients compared to healthy controls [20]. This is important in identifying patients with opportunity to intervene, as stiffness reversal has been demonstrated with increased exercise and lifestyle modification in at risk patients [46,47]. Some studies have shown changes in PWV with medical treatment of hypertension [47–49], but patients with hypertension that are treated to normotension still have elevated arterial stiffness markers [45,50]. Knowing which patients have elevated stiffness even after medical treatment would be instrumental in further risk stratification. There may even be opportunity to investigate which further treatments would improve this population’s stiffness parameters, and therefore cardiovascular risk.

In order to effective monitor patients with cardiovascular risk, speckle tracking as a tool can be integrated into current ultrasound protocols. This should be done by obtaining mid-transverse and longitudinal B-mode recordings of the common carotid artery across the cardiac cycle, and can be done when seeing a patient in clinic without the need for full duplex ultrasound protocol images, such as color Doppler or velocity measurements. Speckle tracking then requires software for post-hoc image analysis, which can be used on standard computers used in the clinical setting. Most evidence suggests a modulus greater than 1 MPa indicative of elevated cardiovascular risk, with values around 0.5 MPa as low risk [23,24,31]. High risk values of circumferential strain have been reported around 2.4%, and normalized to pulse pressure around 4%, while low risk values are reported around 5.5%, and normalized to pulse pressure around 11.4% [23,24]. There is a notable amount of variability in how these values are reported, however, in addition to other stiffness parameters, so further intensive investigation into the values that correspond to an abnormally stiff carotid artery is warranted. Ultimately, we recommend performing this assessment on patients with cardiovascular risk factors that could potentially have subclinical vascular disease, where stiffness parameters would be able to offer insight into the need for aggressive risk factor modification.

## Conclusion

Overall, the evidence supporting the utility of speckle tracking technology in the carotid artery suggests integration into routine vascular ultrasound will aid in the evaluation of treatment of patients at risk of developing vascular disease. This has the potential to influence medical management of systemic disease states such as hypertension or diabetes. Further validation of parameter cutoffs and in-clinic protocol development will increase the ease of adoption and will allow clinicians to better prevent progression of cardiovascular disease.

## Figures and Tables

**Table 1:** Primary literature assessing carotid artery stiffness and how it relates to cardiovascular outcomes.

Study	# of patients	Follow-up	Outcomes	Analysis
Blacher et al.	79	25+/−7 months	Cardiovascular mortality	Kaplan-Meier and log rank analysis in E>1 MPa vs E<1 MPa in ESRD patients
Stork et al.	367	48 months	Cardiovascular mortality	Cox regression, mortality predicted by number of plaques and elastic modulus
Gaszner et al.	250	N/A	Local carotid PWV, PWV	125 CAD vs 125 healthy patients, T-test showed increased PWV and local PWV in patients with CAD
Shroff et al.	55	N/A	Stiffness index	Pearson’s correlation coefficient demonstrated strong relationship between carotid stiffness index and age and vascular inflammatory markers BNP and CRP
Kim et al.	104	N/A	Circumferential strain, strain rate, CIMT	Strain and strain rate significantly lower in patients with CAD
Vriz et al.	92	N/A	Elastic modulus, stiffness index, CIMT	Multivariate analysis demonstrated correlation between carotid stiffness and diastolic heart dysfunction
Catalano et al.	47	N/A	Circumferential strain, CIMT, stiffness index	Compared outcomes between three cardiovascular risk groups, where risk groups were calculated via the CUORE project criteria, strain significantly different between groups
Saito et al.	130	N/A	Circumferential strain, stiffness index, PWV	Compared healthy (n=90) to hypertension (n=40), T-Test shows significantly lower circumferential strain in hypertensive group
Yang et al.	100	N/A	Circumferential strain, strain rate, PWV	Young vs older individuals, segmental strain showed changes in younger group that PWV did not detect
Rosenberg et al.	29	N/A	Circumferential strain, strain rate, stiffness index	18–35 year-old group vs 55–75 year-old group, strain and strain rate were significantly higher in the younger group compared to older group
